# High sensitivity methods for automated rib fracture detection in pediatric radiographs

**DOI:** 10.1038/s41598-024-59077-5

**Published:** 2024-04-10

**Authors:** Jonathan Burkow, Gregory Holste, Jeffrey Otjen, Francisco Perez, Joseph Junewick, Andy Zbojniewicz, Erin Romberg, Sarah Menashe, Jamie Frost, Adam Alessio

**Affiliations:** 1 Michigan State University, East Lansing, MI 48823 USA; 2 Seattle Children’s Hospital, Seattle, WA USA; 3 Helen DeVos Children’s Hospital, Grand Rapids, MI USA; 4 Advanced Radiology Services, Grand Rapids, MI USA

**Keywords:** Diagnosis, Bone imaging, Radiography, Biomedical engineering, Computational science

## Abstract

Rib fractures are highly predictive of non-accidental trauma in children under 3 years old. Rib fracture detection in pediatric radiographs is challenging because fractures can be obliquely oriented to the imaging detector, obfuscated by other structures, incomplete, and non-displaced. Prior studies have shown up to two-thirds of rib fractures may be missed during initial interpretation. In this paper, we implemented methods for improving the sensitivity (i.e. recall) performance for detecting and localizing rib fractures in pediatric chest radiographs to help augment performance of radiology interpretation. These methods adapted two convolutional neural network (CNN) architectures, RetinaNet and YOLOv5, and our previously proposed decision scheme, “avalanche decision”, that dynamically reduces the acceptance threshold for proposed regions in each image. Additionally, we present contributions of using multiple image pre-processing and model ensembling techniques. Using a custom dataset of 1109 pediatric chest radiographs manually labeled by seven pediatric radiologists, we performed 10-fold cross-validation and reported detection performance using several metrics, including F2 score which summarizes precision and recall for high-sensitivity tasks. Our best performing model used three ensembled YOLOv5 models with varied input processing and an avalanche decision scheme, achieving an F2 score of 0.725 ± 0.012. Expert inter-reader performance yielded an F2 score of 0.732. Results demonstrate that our combination of sensitivity-driving methods provides object detector performance approaching the capabilities of expert human readers, suggesting that these methods may provide a viable approach to identify all rib fractures.

## Introduction

Child physical abuse is a serious problem with over 108,000 confirmed cases in the US in 2020, leading to an estimated 1750 deaths (2.38 for every 100,000 children) from abuse and neglect^1^. Bone fractures are the second most common presentation for abused children, after soft-tissue injuries^2^. The most common fracture site in abused children is the ribcage, accounting for over 70% of fractures^3^. In young children, studies have shown rib fractures are a result of child abuse 80–100% of the time^4,5^ and is 23.7 times more likely from abuse than from an accident^6^. In short, rib fractures are extremely important to detect as they are a sentinel injury for physical abuse in young children that portend poor outcomes; a single rib fracture in children is associated with a 2.5 times increase in mortality rate^7^.

Detection of rib fractures in pediatric radiographs is difficult. Expert, specialized radiologists performing their first reads on X-rays can miss up to two-thirds of all present rib fractures^8^. Furthermore, the contraction of the radiology workforce with increasing radiology workloads has led to less time available to scrutinize imaging examinations. With the ever-increasing application of deep learning to the medical field, the implementation of computer vision models as a first-read augmentation technique could improve both detection of rib fractures and speed of interpretation of radiologists, especially for non-expert readers.

Moreover, a driving motivation for our work is to improve the sensitivity of fracture detection to ensure that few to no fractures are missed during interpretation. The pediatric rib fracture detection task warrants methods that are high sensitivity, even if they have reduced precision, because of the high positive-predictive value of rib fractures as an indicator of child abuse and the downstream risk of missing a fracture is considerable^5^. Automatic object detectors with high sensitivity (identical to high recall) have the potential to augment radiologist performance by flagging multiple suspicious regions in the image leading to higher sensitivity interpretations.

In this paper, we present current state-of-the-art performance for the detection of rib fractures on a custom curated data set of pediatric chest radiographs. We expand upon our previous work of using dynamic decision thresholds^9^ and demonstrate the value of ensembling convolutional architectures and applying varied-input processing methods. We also compare model performance with inter-reader variability among board-certified radiologists to substantiate the capabilities of these models versus expert human readers.

## Related work

### Object detection

Object detection performance is rapidly improving thanks to a host of refinements to two-stage and single-stage methods^10^. Two-stage architectures, such as R-CNN^11^, Faster R-CNN^12^, and Mask R-CNN^13^, include region proposal, region classification, and location refinement steps. Alternatively, single-stage architectures, such as Single Shot Detector (SSD)^14^, You Only Look Once (YOLO)^15^, RetinaNet^16^, and TridentNet^17^, feature a single mapping of images to regression outputs defining the boundary of objects. Originally praised and implemented due to their faster real-time inferencing capabilities, single-stage architectures and numerous variants are now becoming more common and achieving results on par with two-stage methods.

### Rib fracture detection

Numerous deep learning methods for rib fracture detection have been developed in the last 5 years for volumetric CT images^18–20^. Yao et al. implemented a three-stage process for rib fracture detection, beginning with a U-Net for bone segmentation of the CT image, isolating the ribs and removing additional bony structures such as scapulae, and classifying whether a fracture was present via a 3D DenseNet^21^. A similar approach was taken by Zhang et al., utilizing a nnU-Net to segment areas of ribs that may contain a fracture and a secondary stage with a DenseNet to classify the segmented region^22^. MICCAI hosted the RibFrac challenge in 2020 that invited methods to detect the location and classify fractures into four clinical categories on CT images^23^. The three leading methods from this challenge used RetinaNet or variations of masked R-CNNs. Fewer methods have been developed for rib fracture detection on 2D radiographs.

There have been substantial efforts to apply deep learning to chest X-ray images, thanks partially to large publicly available data sets of common chest pathologies^24,25^. These efforts largely focus on classification of the image and on lung diseases such as fibrosis, pneumonia, and COVID-19^26^. There have been successful efforts for improved wrist fracture detection on radiographs^27^, but there has been less effort focused on rib fracture assessment^28^. Gao et al. performed rib fracture detection on radiographs with their proposed CCE-Net where multiple feature extraction modules were fused together as inputs to a two-stage detection network demonstrating improved performance compared to competing R-CNN and YOLOv4 architectures^29^. To our knowledge, there are no published methods attempting to automatically detect the location of rib fractures on pediatric chest X-rays.

Detection of rib fractures on pediatric chest radiographs is challenging for a host of reasons: complex anatomy of the ribs, age-related variations of the rib structure, rib fracture location and orientation, and perceptional difficulties from overlying anatomy and artifacts (e.g., monitor leads, support devices, clothing)^30^. The age of fractures also make identification difficult. In children, acute rib fractures may be undetectable on chest radiographs and in some cases only become evident after callus formation (new bone growth) develops 10–14 days into the healing process^31^. It is therefore not surprising that missed rib fractures in children are quite common and that the sensitivity for detection of any rib fracture on pediatric chest radiography by experts is only about 31%^32,33^.

We have previously presented preliminary results in support of pediatric rib fracture detection. In one effort, we developed a method for chest segmentation tailored for pediatric radiographs^34^ that serves as a pre-processing step in this current work. Likewise, we proposed a sensitivity driving approach named “avalanche decision schemes”^9^. This current work expands on our prior efforts with the following innovations: (1) use of a larger custom annotated data set, (2) development of varied-input-processing methods, (3) application of ensembled models, and (4) comparison against expert reader performance for the same task on matched data.

## Methods

**Figure 1:**
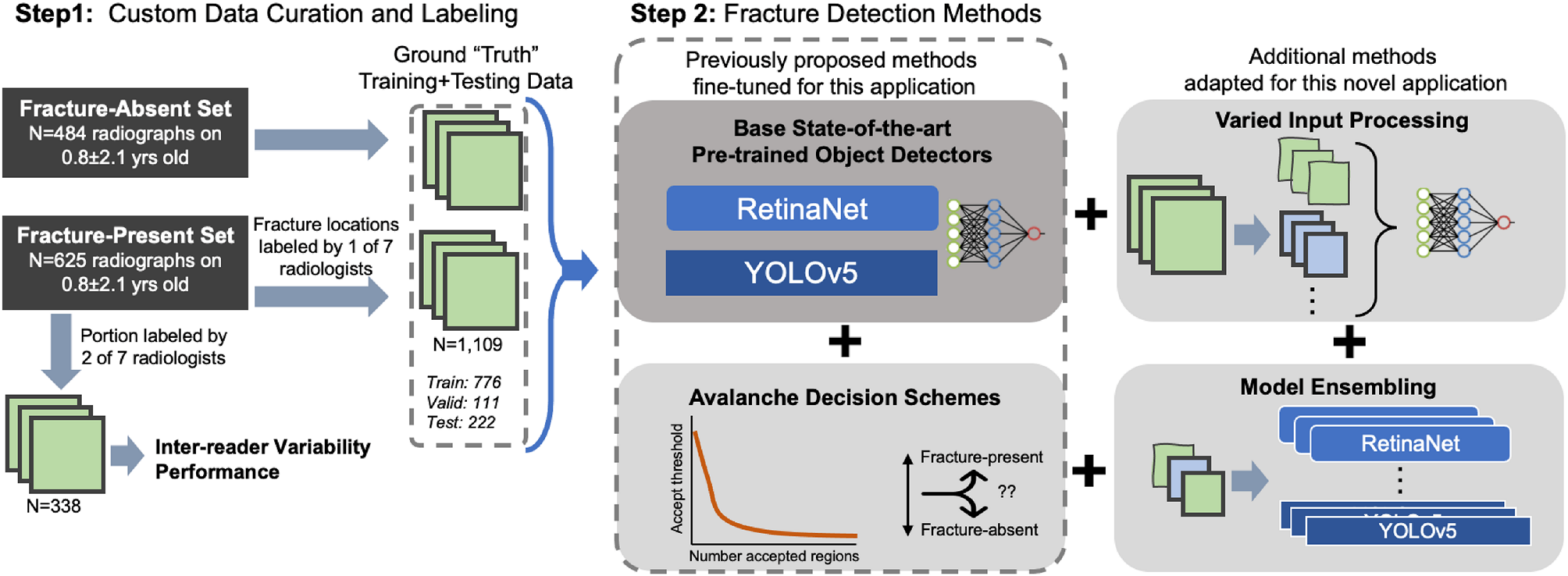
Summary of the major contributions of this paper including curation of a labeled dataset and the addition of up to three high-sensitivity methods (avalanche decision scheme, varied-input processing, and ensembling) to start-of-the-art pre-trained object detectors. In addition, inter-reader variability between expert radiologists was evaluated on 338 of the 624 fracture-present radiographs.

We propose a start-to-finish methodology for detection of rib fractures in pediatric chest radiographs. The pipeline for processing through evaluation for the rib fracture detection task is as follows: original DICOM radiograph file $\rightarrow$ thoracic region segmentation via trained U-Net $\rightarrow$ cropping around segmented region $\rightarrow$ image processing/filtering $\rightarrow$ CNN architecture training and inference $\rightarrow$ evaluation of CNN detection proposals with applied avalanche decision schemes and/or ensembling. Each step in this pipeline is elucidated in the following sections. The major contributions of this work are presented in Fig. 1.

### Dataset curation and labeling

This study was approved by the institutional review board at Seattle Children’s Hospital (STUDY00000853) with informed consent waived due to the study design. Data were collected for this minimal risk retrospective analysis and all methods and experiments in this study were carried out in accordance with relevant guidelines and regulations of the institution (Seattle Children’s Hospital). In this convenience sample, we first searched the medical record for patients with chest radiographs with confirmed rib fractures and identified 624 cases. Gender and age statistics for these fracture-present studies were extracted. An age- and gender-matched sample of chest radiographs with no rib fractures was created. Chest radiographic images were extracted from the medical record and fully anonymized. These images had (height by width) dimensions on average of 2348 ± 685 by 2134 ± 500 pixels with pixel spacing of 0.128 ± 0.023 mm. All images were quantized from 12- or 16-bit integer to 8-bit integer precision and analyzed with their original pixel spacing.

In total, the dataset contains 1109 unique patients, of which 624 are fracture present and 485 are fracture absent. There are 385 ($34.7\%$) female and 724 ($65.3\%$) male patients. The average age of patients is $281.74 \pm 769.42$ (range 0–7300; median 84; IQR 224) days. In order to perform and evaluate object detection, we obtained hand-drawn ground-truth annotations for all images. In short, the fracture present cases were each interpreted by one of seven board-certified pediatric radiologists with 5–20 years of experience. During interpretation, the radiologists had access to all of the available radiographic views of the chest (usually supine anterior–posterior (AP) although occasionally other views were available). They were instructed to draw bounding boxes as closely around each detected fracture as possible on the AP view only; the object detection methods discussed below were only applied to the AP view image. Of the total 624 fracture-present images, 338 were read by two board-certified pediatric radiologists to enable estimation of inter-reader variability. This inter-reader variability estimate served as a performance baseline for evaluating the proposed methods.

### U-Net segmentation and cropping

**Figure 2:**
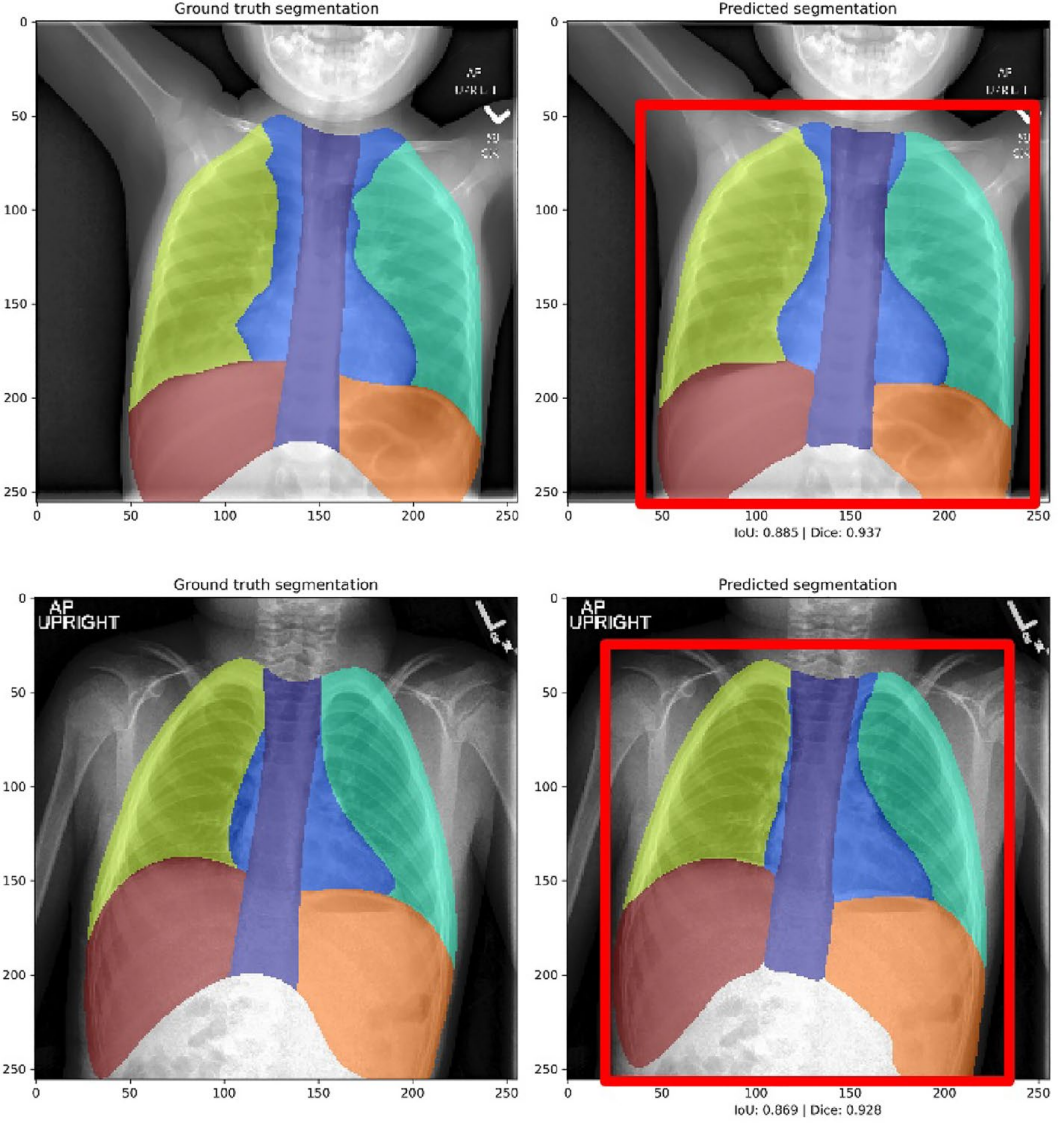
Representative results from multi-class segmentation showing manually labeled images (left) and U-Net results (right) with final automatically-generated cropped region represented by red box.

In our prior work^34^, we trained a U-Net model^35^ to segment chest radiographs into multiple anatomic regions. Here, we improved that effort with the use of a more advanced U-Net3+ architecture that includes full-scale skip connections^36^. Labels for training were manually drawn to segment the chest into seven non-overlapping regions: left and right lung, left and right “subdiaphragm” (the thorax below the superior boundary of diaphragm), spine, mediastinum, and background. In total, users manually labeled 469 radiographs. After each inference from the U-Net3+ model, the proposed segmentation maps are automatically refined with basic morphological operations to remove small spurious disconnected regions from background and close all foreground regions. Of the 469 labeled images, the U-Net model was trained with 422 and tested on the remaining 47 images. Representative segmentations from the test set are presented in Fig. 2. The mean Dice coefficient for each of the seven regions exceeded 0.88 and visual assessment confirmed that all final cropped images in the test set contained only the thoracic cavity. For this current work, we use this U-Net segmentation to provide a tight cropping window around the region of the image containing ribs and for the varied input processing ensembles described below.

### Deep learning models

For RetinaNet, we used the ResNet-50 backbone with pre-trained weights on ImageNet^37^. We trained all RetinaNet models on our dataset using a NVIDIA V100S for a maximum of 300 epochs at a batch size of 8 using the Adam optimizer. The learning rate was set initially to 0.0001 and was decreased by one-tenth if validation performance did not improve within 4 epochs. The dataset was augmented with a $50\%$ chance of applying any of the following transformations to each image: shift/scale/rotate, horizontal flip, random brightness, random contrast, or Gaussian blur. Training would cease via an early stopping clause if performance on the validation set had not improved in 30 epochs.

We also utilized Ultralytics’ open-source YOLOv5 repository^38^, using the large L6 model pre-trained on the COCO dataset prior to training on our dataset. Similarly, all YOLOv5 models were trained on a NVIDIA V100S for a max of 300 epochs and batch size of 8. A stochastic gradient descent (SGD) optimizer was used with momentum 0.937 and weight decay of 0.0005. Learning rate was initially 0.01 and decreased linearly each epoch. There was also an early stopping feature that stopped training if no improvement in validation was observed after 100 epochs.

### Avalanche decision schemes

Our preliminary work proposed adjusting the decision threshold for fracture positive proposals as a function of the number of already accepted bounding box proposals^9^. The decision threshold is not fixed, but rather changes depending on the number of high probability proposed regions. This approach is motivated by the reality that if a subject has one fracture they are very likely to have more than one fracture, and a subject with two fractures is very likely to have three, and so on. These likelihoods presented in Table 1 are updated using the now larger dataset than our preliminary work. In this table, the first row shows that 444 images in the training set have at least 1 fracture; If an image has at least one fracture, there is a $73.6\%$ likelihood of having more than one fracture. Similarly, if an image has at least 2 fractures, there is a $81.3\%$ likelihood of having more than 2 fractures.

**Figure 3:**
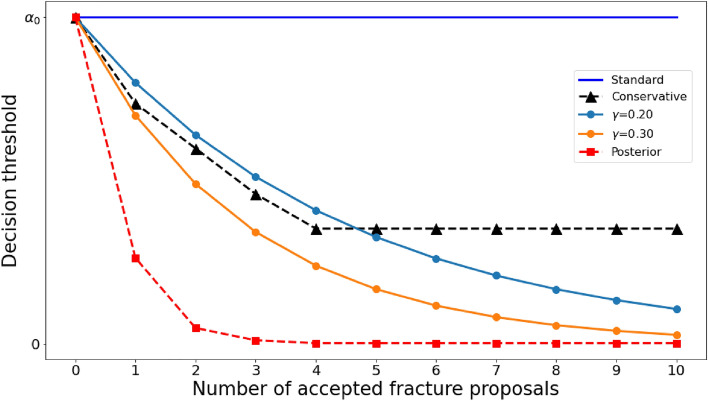
Plot of relative decision threshold for bounding box acceptance as a function of the number of accepted proposals.

We explored different relationships for decision thresholds versus apparent number of accepted fractures as presented in Fig. 3. For the “Standard” approach the decision threshold is constant no matter how many proposed regions clear this threshold level, and typically the threshold is set at 0.5 (all proposals with confidence greater than $50\%$ are accepted). For the avalanche approaches, the decision threshold decreases as more proposed regions are accepted. If *n* regions have a probability greater than $a_{n-1}$, then the new threshold is set to $a_n = r \cdot a_{n-1}$. We evaluated different schemes for setting the reduction, *r*. Specifically, the “Posterior” method uses threshold reductions based on the likelihood information presented in Table 1, with $r=(1-\mathbb {P}(X>x|X\ge x)) \approx 0.25$ for $n = [1,2,3,4]$. The “Conservative” method uses $r\approx 0.75$ for $n = [1,2,3,4]$. The other methods, labeled with “$\gamma$”, fixed the reduction to $r=(1-\gamma )$ for all *n*. The values for $\gamma$ were selected based on initial testing of multiple values ranging from 0.05 to 0.5 in increments of 0.05; please see preliminary work for additional details^9^.

**Table 1:** Posterior likelihood for the training dataset, with each row summarizing probabilities of having more fractures, *X* (a random variable), when at least *x*=1, 2, 3, and 4 fractures are present (a deterministic variable).

X $\ge$ x Fractures	*N*	$\mathbb {P}$(X>x|X$\ge$ x)
X $\ge$ 1	444	$73.6\%$
X $\ge$ 2	327	$81.3\%$
X $\ge$ 3	266	$76.7\%$
X $\ge$ 4	204	$77.0\%$

We improved on our previous work by implementing a non-maximum suppression (NMS) step, a common approach to filter out regions proposals with a large overlap of each other. This NMS step is applied after the avalanche decision schemes have been applied on the given trained model predictions. This is particularly effective on networks like RetinaNet where the number of bounding box proposals per image is significantly higher than more reserved models such as YOLOv5.

### Input processing

**Figure 4:**
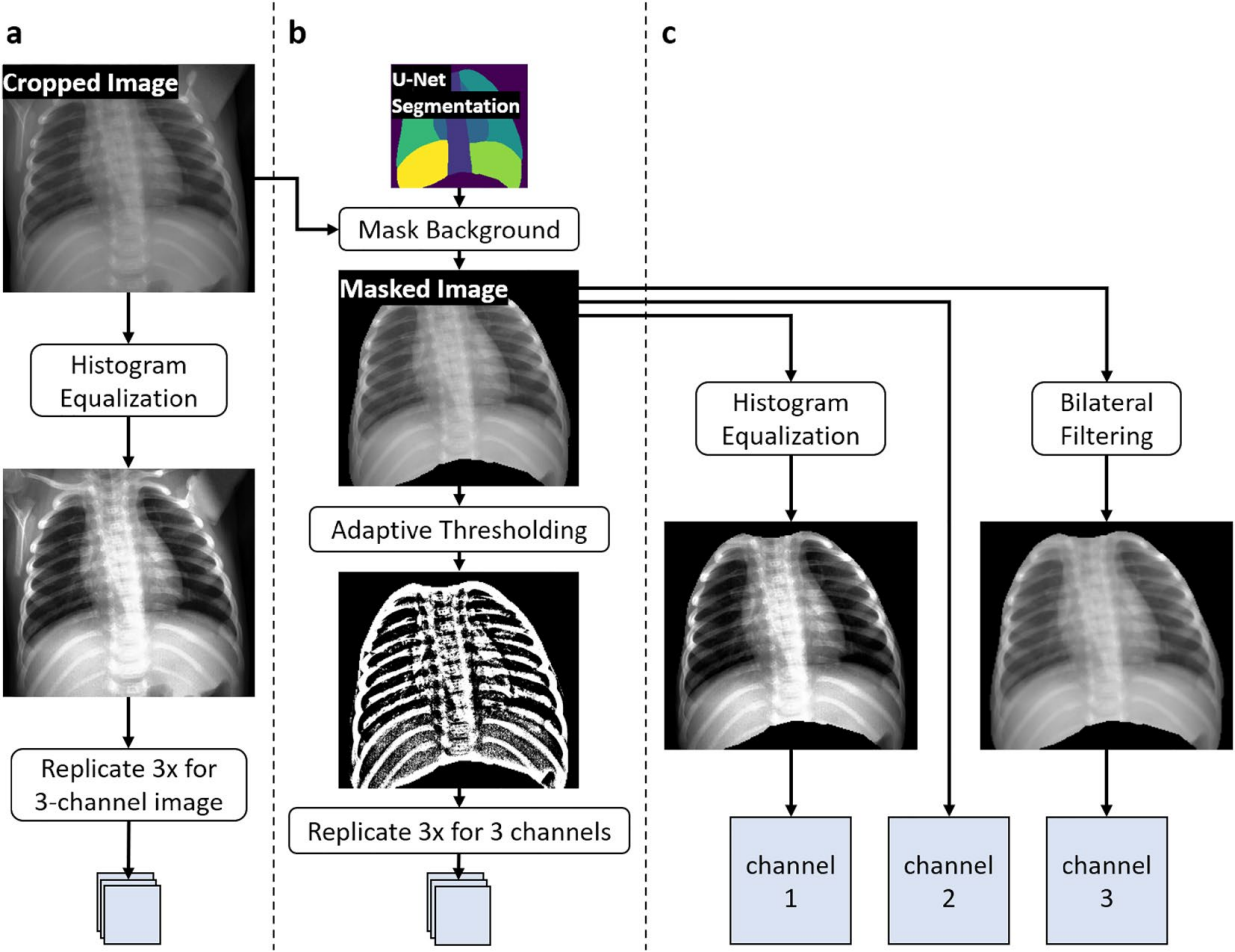
The three types of image processing used in the varied-input-processing ensemble models. (**a**) normal-cropped histogram equalized 3x-stacked images; (**b**) segmentation-masked adaptive thresholding 3x-stacked images (binary); (**c**) segmentation-masked raw, histogram equalized, and bilateral low-pass filtered (blended).

We wanted to explore how varying the type of processing performed on the input images changed the performance of the trained models. All types of processing were applied following the segmentation and cropping via the U-Net discussed above. Figure 4 provides a visualization of the different types of processing and how they are combined to create input images for training and evaluation. Method **a** applies histogram equalization to the single-channel, grayscale image array after which the array is replicated three times to provide the three channel input to the object detector models.

The two additional variations go an additional step by utilizing the pixel-level segmentation information from the U-Net from the previous pre-processing stage. After cropping the image around the segmented thoracic region, all background pixels are masked out to generate a masked foreground image containing only anatomical structures. In method **b**, adaptive thresholding is applied using a Gaussian weighting method to determine threshold values in a given neighborhood of pixels. This transforms the image from grayscale to binary, with 1 (white) representing pixels above the threshold and 0 (black) below, providing a rough segmentation of just the ribs. This binary mask is then stacked three times as the final image.

In method **c**, the masked image goes through two separate filtering operations, inspired by Heidari et al.^39^: histogram equalization (like method **a**) for increased contrast and bilateral low-pass filtering for edge-preserving noise reduction. The low-pass filter uses mid-line $\sigma$-space and range values of 150, with a 9 pixel neighborhood diameter. The original masked image, histogram equalized masked image, and bilateral filtered masked image are then stacked as the three channels for the detector input, which we label as “blended” input.

### Ensembles

We also investigated the impact of model ensembles on rib fracture detection. Model ensembles with deep neural networks have shown better generalizability as well as improved performance on tasks with smaller datasets^40–42^. To survey this, we tested the following ensembles:

1. Same-Model Ensemble: The simplest form of ensembling is the combination the proposal results of multiple identical models each with different training runs initialized with different seeds, similar to the deep ensembles analyzed by Lakshminarayanan et al.^43^.
2. Hybrid-Model Ensemble: This is a slight variation to the same-model ensemble, combining an equal number of training runs of both deep learning architectures we tested; for example, combining one run of RetinaNet with one run of YOLOv5.
3. Varied-Input-Processing Ensemble: The final type of ensembling models incorporated all three of the different image pre-processing operations as summarized in Fig. 4, requiring at minimum three trained models trained on each of the input processing variations.

Prior to final evaluation, proposed bounding boxes from all members of each ensemble were aggregated together and overlapped boxes then removed via non-maximum suppression (NMS) with an intersection-over-union (IOU) threshold of 0.45. This threshold was set based on initial validation experiments, but not fully optimized across all model variants.

### Training and evaluation

Twenty percent of the total dataset were withheld as the fixed test set (N = 222 images), with half randomly drawn from fracture-present images and the other half randomly drawn from fracture-absent images. The remaining $80\%$ of data was then used to create the training ($70\%$) + validation ($10\%$) sets. All evaluations were performed after training with a 10-fold cross-validation strategy in order to examine the range in model performance; the ten separate training and validation sets were randomly drawn with replacement between each set.

Object detection performance was evaluated in terms of recall, precision, and F2 score on the fixed test set. An intersection-over-union (IOU) threshold of 0.30 was applied across all model and ensemble evaluations to identify concordance between model predictions and labeled annotations. Supplementary Fig. S3 provides rationale for the selection of this IOU threshold. We used F2 score rather than F1 to give recall/sensitivity twice the importance of precision, considering this task warrants high sensitivity performance as discussed above. Max F2 scores are also provided for each combination by finding the highest F2 score achieved across all potential decision thresholds. Furthermore, to summarize average performance across a range of settings, we calculated mean average precision (mAP) by computing the areas under multiple precision-recall curves generated at IOU thresholds ranging from 0.25 to 0.75 in 0.05 increments. Note that mAP was not be calculated for the avalanche decision schemes since precision-recall curves are not analogous between fixed and dynamic decision thresholds.

For single-model calculations, we evaluated performance metrics for all ten trained models (trained on each of the ten folds) and report the average ± standard deviation across these models. For two-, three-, and six-model ensembles, twenty ensemble combinations were arbitrarily selected from the multitude of different ways to combine 2, 3, or 6 models from the 10-fold data; In other words, twenty model combinations were taken from the 10 choose 2 (45), 10 choose 3 (120), and 10 choose 6 (210) possible combinations, respectively, that were then evaluated and averaged.

## Results

**Figure 5:**
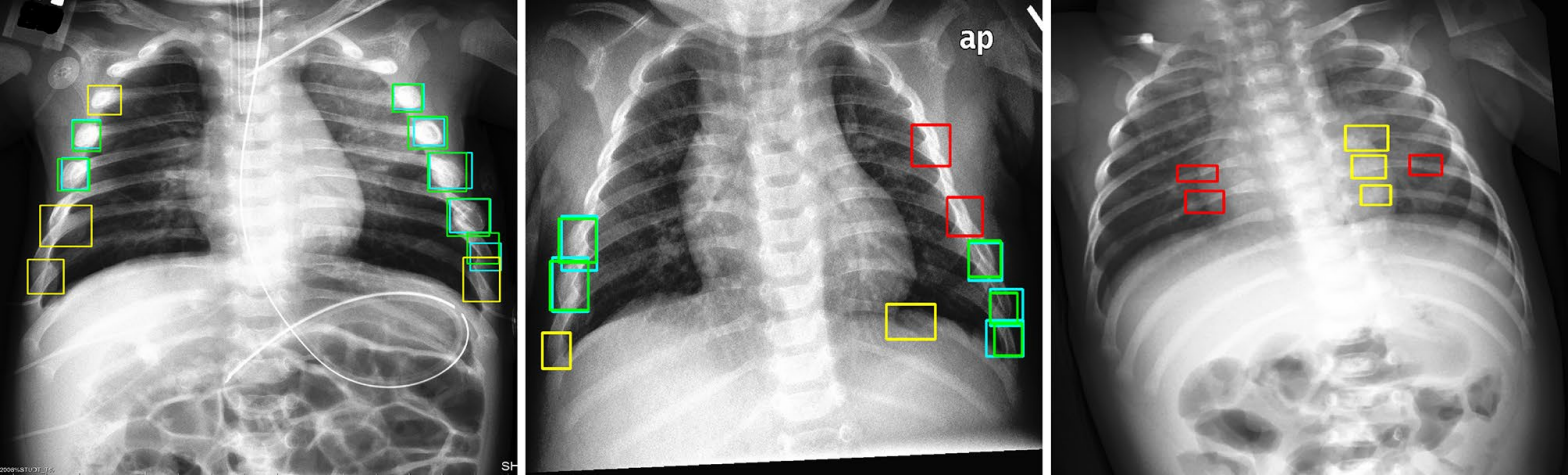
Test set images with ground truth (teal, red) and model predictions (green, yellow), with true positives (green), false positives (yellow), and false negatives (red). Predictions from the 6x-YOLOv5 ensemble trained on histogram equalized input images with a $\gamma =0.20$ avalanche scheme, achieving $0.536 \pm 0.044$ precision, $0.795 \pm 0.022$ recall, and $0.723 \pm 0.010$ F2 score.

Representative test set images with ground truth annotations and model predictions are presented in Fig. 5.

### Inter-reader variability

Of the total 624 fracture-present images, 338 were read by two board-certified radiologists. We calculated inter-reader performance for two different data sets: (1) Images from the test set (which contains 222 images, although only 111 of these are fracture-present and therefore interpreted by radiologists), and (2) Images from the set of 338 fracture-present images that have been read by two radiologists. For clarity, this inter-reader study was performed on fracture-present only images, while the deep learning training and testing was performed on present and absent images.

On the test set, the first reader marked 536 total rib fractures for an average of $4.83 \pm 3.30$ (range 1–14; median 4; IQR 5) fractures per image. The second reader marked 486 fractures overall, averaging $4.38 \pm 3.74$ (range 1–27; median 4; IQR 4) fractures per image. Setting the first reader’s annotations as “ground truth” between the two, fractures were scored as true positive (second reader box matches a reader 1 box), false positive (reader 2 box has no corresponding reader 1 box), or false negative (reader 1 box has no matching reader 2 box), dictated by an intersection-over-union (IOU) threshold of 0.30.

Three-hundred eighty-five fractures were counted as true positive matches, with 101 false positives and 151 false negatives. This led to the second reader scoring a precision of 0.792, recall of 0.718, and F2 score of 0.732. Essentially, the second reader “detected” just under $72\%$ of the rib fractures discovered by the first reader. With these scores, the second reader’s boxes overlapped reader 1 on average by $84\%$ with a mean intersection-over-union of 0.63 across the 111 images. For clarity, overlapping represents the percentage of reader 1’s annotated box pixels that are covered by the pixels from reader 2’s matching box.

Inter-reader performance metrics remained very similar when looking at all 338 multi-read images. Reader 1 marked 1719 fractures, averaging $5.09 \pm 4.30$ (range 1–22; median 4; IQR 5) fractures per image and reader 2 marked 1567 fractures for an average of $4.64 \pm 4.08$ (range 1–27; median 4; IQR 4) fractures per image. Percent overlap and IOU remained essentially identical at $84\%$ and 0.62. Precision, recall, and F2 score all decreased slightly to 0.777, 0.709, and 0.721. If we were to assume the first reader caught all fractures during their reads, the second reader was able to find $71\%$ of the fractures in their reads. This again leaves over one-quarter of all fractures undetected between expert radiologists.

### Base network performance

Base network performance was evaluated for single-model performance of either RetinaNet or YOLOv5 using histogram equalization image pre-processing (method (**a**) from Fig. 4). These results are presented in the Standard rows in Table 2 (and below with the nomenclature 1x-R^a^ and 1x-Y^a^). RetinaNet achieved $0.892 \pm 0.015$ precision, $0.430 \pm 0.014$ recall, and $0.480 \pm 0.014$ F2 score, whereas YOLOv5 scored $0.897 \pm 0.032$ precision, $0.434 \pm 0.040$ recall, and $0.484 \pm 0.037$ F2 score. When compared to expert-level human performance, both networks had marked higher values in precision but lower recall and therefore F2 scores. If either network were to predict a region for a potential rib fracture, they were essentially $90\%$ likely to be correct in that prediction. However, both networks detected less than half of all rib fractures in the test set.

### Avalanche decision

Table 2 presents results with the avalanche schemes applied to single RetinaNet and YOLOv5 models trained on the histogram equalized inputs. The posterior scheme with RetinaNet reduced precision to $0.141 \pm 0.015$, a $84.19\%$ decrease, whereas recall increased $102.8\%$ to $0.872 \pm 0.013$. This, however, lead to an F2 score of $0.427 \pm 0.026$ which is $11\%$ lower than standard. The best performing avalanche scheme for RetinaNet was the conservative scheme, where the $40.6\%$ reduction in precision and $69.8\%$ increase in recall saw the F2 score increase to $0.679 \pm 0.010$ ($+41.5\%$).

**Table 2:** RetinaNet and YOLOv5 single-model results comparing performance with the standard fixed decision threshold and applying the various avalanche schemes. $\gamma$ represents the constant rate reduction between each decision threshold in the avalanche scheme.

Model	Scheme	Precision	Recall	F2
RetinaNet	Standard	**0.892** ± 0.015	0.430 ± 0.014	0.480 ± 0.014
	Posterior	0.141 ± 0.015	**0.872** ± 0.013	0.427 ± 0.026
	Conservative	0.530 ± 0.023	0.730 ± 0.015	**0.679** ± 0.010
	$\gamma =0.15$	0.304 ± 0.028	0.766 ± 0.015	0.586 ± 0.019
	$\gamma =0.20$	0.256 ± 0.023	0.770 ± 0.024	0.548 ± 0.015
YOLOv5	Standard	**0.897** ± 0.032	0.434 ± 0.040	0.484 ± 0.037
	Posterior	0.759 ± 0.164	**0.647** ± 0.101	**0.652** ± 0.051
	Conservative	0.831 ± 0.101	0.590 ± 0.075	0.622 ± 0.053
	$\gamma =0.15$	0.814 ± 0.120	0.587 ± 0.077	0.615 ± 0.050
	$\gamma =0.20$	0.816 ± 0.114	0.593 ± 0.087	0.621 ± 0.060

Bolded values highlight the highest value for each metric for each model.

Interestingly, YOLOv5 had a relatively minor decrease in precision but marked improvement in recall, and therefore F2 score, with the avalanche decision schemes. Precision decreased by 7–15% across the schemes while recall increased between 35–49%. As a result, the lowest performing YOLOv5 model with an avalanche scheme had an F2 score of $0.615 \pm 0.050$ and was still $27.1\%$ better than standard; the best performance came from the posterior scheme with an F2 score of $0.652 \pm 0.051$ ($+34.7\%$). Considering the large number of different avalanche schemes, the remaining results will only present schemes corresponding to best F2 score performance for each model or ensemble.

### Combining avalanching, input processing, and ensembling

**Figure 6:**
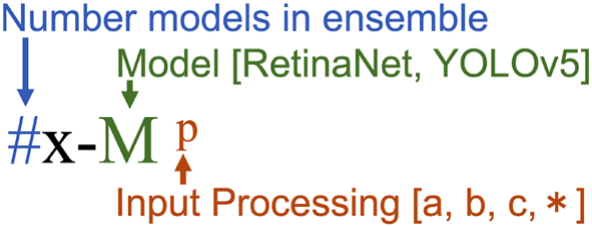
Explanation of model nomenclature for ensembling combined with different input processing. The selection of input processing [a,b,c] is described in Fig. 4 and varied input processing [*] uses one from each input processing type. For example, results presented for method 3x-R* would be for an ensemble of three RetinaNet models (trained on different folds) using varied input processing.

Performance of combining methods are presented in Tables 3 and 4, with the former including evaluations only with the standard decision scheme (i.e., fixed acceptance threshold of 0.50 for all model predictions) and the latter including the best avalanche scheme for each model and/or ensemble. Table 3 also includes inter-reader variability performance at the top for comparison between expert human readers and deep learning models. For full results of all models and ensembles, see Supplementary Tables S1 and S2. An explanation of the model nomenclature is presented in Fig. 6.

Changing from the histogram equalized inputs to the two alternative image processing methods ((**b**) binary and (**c**) blended) slightly altered performance for both base networks in different ways. RetinaNet with binary inputs (1x-R^b^) incurred a small decrease in precision to $0.852 \pm 0.015$ ($-4.5\%$) and large decreases in recall and F2 score at $0.344 \pm 0.027$ ($-18.75\%$) and $0.390 \pm 0.028$ ($-20\%$), respectively. Blended inputs (1x-R^c^) also resulted in a 1.01–1.25% decrease in all three measures compared with method (**a**). YOLOv5 had a similar decrease in performance using the binary inputs, with 1x-Y^b^ achieving $0.872 \pm 0.060$ ($-2.79\%$) precision, $0.320 \pm 0.049$ ($-26.27\%$) recall, and $0.365 \pm 0.050$ ($-24.59\%$) F2 score. However, the blended method (**c**) for YOLOv5 led to the best single-model (non-ensembled) recall and F2 performance of all variants: 1x-Y^c^ scored $0.880 \pm 0.024$ ($-1.9\%$) precision, $0.464 \pm 0.043$ ($+6.91\%$) recall, and $0.512 \pm 0.041$ ($+5.79\%$) F2 score. As presented below, the YOLOv5 architectures using the varied inputs (ensembles of models using input processing a, b, and c) had improvement over the other processing methods.

Using the standard decision scheme, the four three-model ensembles, 3x-R^c^, 3x-R*, 3x-Y^c^, and 3x-Y*, performed very similarly with regards to precision, scoring within $0.6\%$ of one another. Compared to their single-model versions, ensemble methods resulted in improved recall and thus F2 scores. While the best single-model recall was $0.464 \pm 0.043$, the worst performing three-model ensemble (3x-R*) achieved $0.523 \pm 0.014$ ($+12.7\%$) and the best performing ensemble (3x-Y^c^) reached $0.599 \pm 0.021$ ($+29.1\%$). This led to an F2 score of $0.633 \pm 0.018$ with the 3x-Y^c^ model. The mean average precision (mAP) trended upward as ensemble size increased and had similar trends as the F2 score, demonstrating that these models have similar rankings in performance across a range of inference hyper-parameters.

After applying avalanche decision schemes, we saw the expected decrease in precision and increase in recall. The 3x-Y^c^ ensemble with the $\gamma =0.20$ decision scheme had the superior performance among three-model ensembles achieving $0.725 \pm 0.012$ F2 score, which is within $1\%$ of expert human-level performance. One interesting thing to note is that the three-model ensembles with standard decision schemes have lower F2 scores than single-models with avalanche schemes at the trade-off of maintaining much higher precision values that exceed the inter-reader performance.

**Table 3:** Performance results of selected models with standard decision threshold. I.R.V. represents inter-reader variability performance between two radiologists. Bolded values represent the top two scores for each metric. Superscripts a, b, and c represent the type of input processing to train the models as shown in Fig. 4. Ensembles with * have hybrid inputs, i.e., each ensemble member was trained on a different input processing method.

Models	Precision	Recall	F2	Max F2	mAP
I.R.V. (Test set)	0.792	0.718	0.732	–	–
I.R.V.	0.777	0.709	0.721	–	–
1x-R^a^	**0.892** ± 0.015	0.430 ± 0.014	0.480 ± 0.014	0.630 ± 0.011	0.480 ± 0.008
1x-Y^c^	**0.880** ± 0.024	0.464 ± 0.043	0.512 ± 0.041	0.644 ± 0.046	0.555 ± 0.016
3x-R*	0.812 ± 0.011	0.523 ± 0.014	0.563 ± 0.013	0.649 ± 0.006	0.493 ± 0.008
3x-Y^c^	0.814 ± 0.017	0.599 ± 0.021	0.633 ± 0.018	**0.694** ± 0.008	**0.559** ± 0.006
6x-R^c^	0.762 ± 0.008	0.571 ± 0.009	0.601 ± 0.008	0.666 ± 0.004	0.499 ± 0.004
6x-Y^c^	0.756 ± 0.010	**0.653** ± 0.008	**0.671** ± 0.007	**0.699** ± 0.006	**0.555** ± 0.004
3x-R*+3x-Y*	0.752 ± 0.019	**0.625** ± 0.021	**0.647** ± 0.017	0.686 ± 0.008	0.522 ± 0.004

**Table 4:** Performance of models from Table 3 with their best corresponding avalanche decision scheme result with respect to F2 score. Bolded values represent the top two scores for each metric. Superscripts a, b, and c represent the type of input processing to train the models as shown in Fig. 4. Ensembles with * have hybrid inputs, i.e., each ensemble member was trained on a different input processing method.

Models	Avalanche	Precision	Recall	F2	Max F2
1x-R^a^	Conservative	0.530 ± 0.023	0.730 ± 0.015	0.679 ± 0.010	0.897 ± 0.054
1x-Y^c^	Posterior	**0.645** ± 0.130	0.724 ± 0.085	0.695 ± 0.041	0.812 ± 0.056
3x-R*	Conservative	0.340 ± 0.013	0.809 ± 0.009	0.634 ± 0.011	**0.898** ± 0.026
3x-Y^c^	$\gamma =0.20$	**0.573** ± 0.058	0.780 ± 0.030	**0.725** ± 0.012	0.812 ± 0.036
6x-R^c^	Conservative	0.311 ± 0.005	**0.816** ± 0.005	0.616 ± 0.005	**0.912** ± 0.025
6x-Y^a^	$\gamma =0.20$	0.536 ± 0.044	0.795 ± 0.022	**0.723** ± 0.010	0.797 ± 0.016
3x-R*+3x-Y*	Conservative	0.314 ± 0.015	**0.841** ± 0.014	0.630 ± 0.011	0.833 ± 0.052

Six-model ensembles with standard decision schemes have lower precision scores than prior model and ensemble sizes, though still being on-par with expert-level performance. Once again, YOLOv5 with the blended, method (**c**) input images achieved the highest F2 score at $0.671 \pm 0.007$, a $6\%$ improvement over its three-model variant. Incorporating avalanche schemes with 3x-Y^c^ utilizing the $\gamma$ avalanche scheme with a fixed rate of 0.20 provided the highest F2 score of $0.725 \pm 0.012$. The most complex 3x-R*+3x-Y* model was unable to achieve the highest performance in any metric, and in fact even had a slightly lower mAP score than the 1x-Y^c^ models, though its recall and F2 performance were still second-highest among the standard decision threshold models/ensembles.

### Max F2 score between standard and avalanche schemes

**Figure 7:**
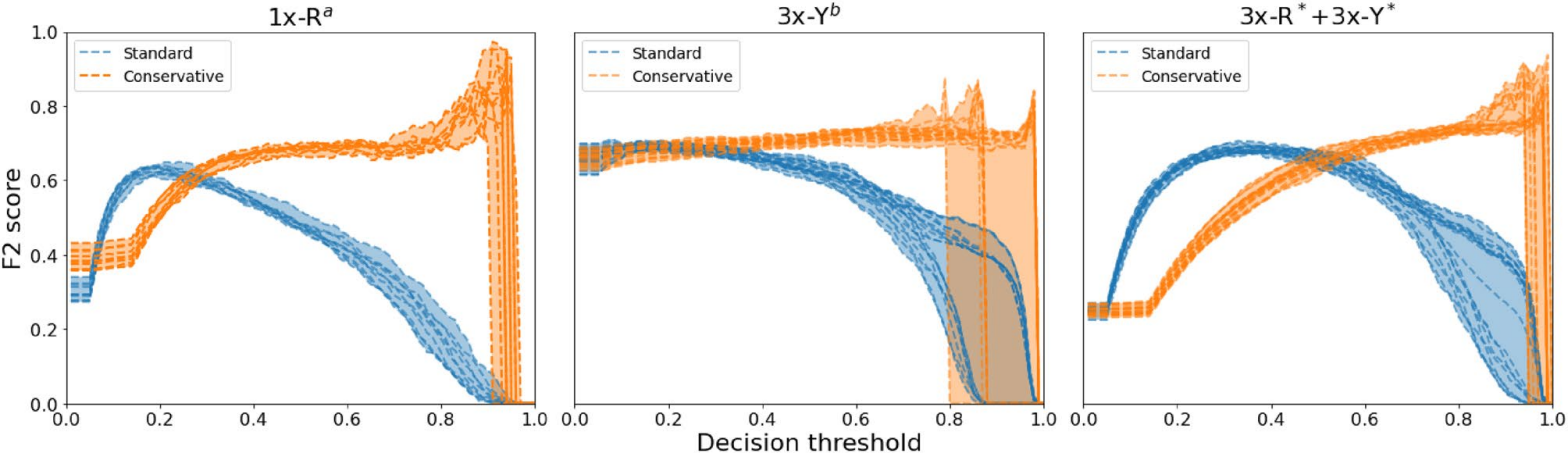
F2 scores across all possible confidence thresholds for 1x-R^a^ models, 3x-Y^c^ ensembles, and the hybrid, 3x-R* + 3x-Y* ensemble. Each dashed line represents performance from one model or combination of ensembles. The best performing avalanche scheme (‘Conservative’) is compared to the ’Standard’ decision scheme. Generally, the avalanche scheme performed better than conventional inferencing, except for low decision thresholds. In every case, the avalanche decision scheme reaches higher max F2 scores than standard.

In order to get a better understanding of how well the avalanche schemes perform compared to the standard inferencing technique, we plotted the F2 scores of a handful of test cases across all possible decision threshold values in Fig. 7. For the avalanche methods, the x-axis of these plots represents the starting threshold ($a_0$) that is then potentially reduced if proposed regions have probabilities greater than $a_0$.

We chose one model from the single-model group: the single RetinaNet using the histogram equalized images. Then we chose the best three-member ensemble with the 3x-Y^c^ ensemble, and the most diverse ensemble with the six-member 3x-R*+3x-Y* ensemble. Each of their best corresponding avalanche decision schemes was plotted along with their traditional decision scheme performances, . In the 1x-R^a^ and 3x-Y^c^ cases, the avalanche scheme performs better than the standard decision scheme across a majority of the possible decision thresholds. For the 3x-R*+3x-Y* ensembles, the standard scheme outperforms the avalanche scheme for thresholds less than around 0.50. In all three cases, not only do the avalanche schemes generally perform better than their standard decision scheme counterparts, but the maximum F2 scores were also higher than what the standard decision versions could attain. This maximum F2 performance can also be seen in the last column of Tables 3 and 4. For each model and/or ensemble, the Max F2 value of its corresponding avalanche scheme is significantly higher than the standard schemes, many reaching a score above 0.9.

## Limitations and future work

This work demonstrated the value of ensembling models to increase recall. We acknowledge that there could be multiple ways to combine model results. In this effort, we only explored combining model results with non-maximum suppression (NMS) of all proposed boxes. This is an affirmative approach that inherently will only result in equal or better recall (coupled with equal or worse precision). Future work could explore additional methods for merging model results.

This work uses a relatively small dataset from a single institution; future efforts are needed to replicate these results and ensure generalizability in larger, more diverse datasets. Moreover, our 624 fracture-present images were labeled with single reads and therefore our fracture-present labels are noisy and likely contain errors. This is especially likely considering the challenge of detecting pediatric fractures and given that our inter-reader variability sub-study showed that along with other measures, the recall between radiologists was just under $71\%$ demonstrating over one-quarter of all fractures were missed by the second reader. Future work using consensus interpretation is needed to improve our labels. Finally, the performance was evaluated on a test set with half fracture-present cases and half fracture-absent cases. While the prevalence of rib fractures in real-world clinical settings will vary depending on the site and nature of the practice (out-patient versus emergency room setting, etc.), this $50\%$ prevalence test set contains a higher likelihood of fractures than would be encountered in practice. Future work is needed to determine performance in realistic clinical settings with more thorough comparisons to expert human performance. Furthermore, future work is needed to evaluate the proposed methods as an in-line augmentation strategy with a radiologist serving as the arbitrator of model predictions; these types of future evaluations are critical for determining the ultimate clinical impact of AI-assisted and AI-augmented interpretation tools.

## Conclusion

We demonstrate multiple methods that improve the sensitivity (i.e. recall) performance of two state-of-the-art object detectors on a custom curated dataset of pediatric chest radiographs. This includes an improvement to our novel dynamic decision threshold avalanche scheme as well as three methods of pre-processing the images. Additionally, various ensembling approaches combined with the aforementioned techniques were investigated. These techniques provided reduced precision with higher recall resulting in improvements in F2 score by extension. Simple ensembles, such as same-model three- and six-model ensembles, offered straightforward improvements over single-model detectors. This is likely due to the enhanced generalizability by training each ensemble member on different cross-validation folds of the training and validation data sets. Interestingly, many of the best performing models utilized the blended method (**c**) of pre-processing, where each channel of the input images was processed differently. The method with the highest F2 score was an ensemble of three YOLOv5 models using the input (**c**) pre-processing and with the $\gamma =0.20$ avalanche scheme. This model achieved an F2 score of 0.725 ± 0.012, which was only approximately $1\%$ below the inter-reader F2 score of expert radiologists of 0.732 and with a recall score exceeding the experts at $0.780\pm 0.030$ versus 0.718. Overall, this work demonstrates promising methods for high sensitivity rib fracture detection that could serve as automatic approaches to augment the performance of radiologists performing pediatric chest radiograph interpretation.

### Supplementary Information

Supplementary Information.

## Data Availability

The datasets generated and analysed during the current study are not publicly available due to the limits of the IRB approval founded on concerns of potential risk of reidentification with these images and because it includes data from a vulnerable population (patients< 18 years of age). Data are however available from the corresponding author upon reasonable request and with permission of Seattle Children’s IRB.
