# Supplementary material for: High sensitivity methods for automated rib fracture detection in pediatric radiographs
Source: Sci Rep. 2024 Apr 10;14:8372. doi: 10.1038/s41598-024-59077-5 (PMC11006902; doi:10.1038/s41598-024-59077-5)
Supplement: Supplementary file 1 — Supplementary Information. [file 41598_2024_59077_MOESM1_ESM.pdf]

Supplementary Information

Models	Precision	Recall	F2	Max F2	mAP
1x-R[a]	**0.892** ± 0.015	0.430 ± 0.014	0.480 ± 0.014	0.630 ± 0.011	0.480 ± 0.008
1x-R[b]	0.852 ± 0.015	0.344 ± 0.027	0.390 ± 0.028	0.570 ± 0.015	0.395 ± 0.010
1x-R[c]	0.883 ± 0.013	0.425 ± 0.027	0.474 ± 0.027	**0.635** ± 0.012	0.473 ± 0.009
1x-Y[a]	**0.897** ± 0.032	**0.434** ± 0.040	**0.484** ± 0.037	0.590 ± 0.062	**0.563** ± 0.009
1x-Y[b]	0.872 ± 0.060	0.320 ± 0.049	0.365 ± 0.050	0.539 ± 0.077	0.462 ± 0.035
1x-Y[c]	0.880 ± 0.024	**0.464** ± 0.043	**0.512** ± 0.041	**0.644** ± 0.046	**0.555** ± 0.016
2x-R[a]	**0.859** ± 0.013	0.488 ± 0.014	0.534 ± 0.013	0.651 ± 0.011	0.495 ± 0.005
2x-R[b]	0.803 ± 0.014	0.408 ± 0.026	0.453 ± 0.025	0.587 ± 0.011	0.412 ± 0.009
2x-R[c]	0.847 ± 0.012	0.487 ± 0.025	0.532 ± 0.023	0.652 ± 0.010	0.488 ± 0.007
2x-Y[a]	**0.862** ± 0.032	**0.523** ± 0.027	**0.567** ± 0.024	**0.672** ± 0.022	**0.571** ± 0.015
2x-Y[b]	0.820 ± 0.062	0.414 ± 0.045	0.459 ± 0.042	0.610 ± 0.020	0.468 ± 0.025
2x-Y[c]	0.835 ± 0.017	**0.558** ± 0.024	**0.597** ± 0.021	**0.690** ± 0.012	**0.562** ± 0.012
3x-R[a]	**0.839** ± 0.012	0.516 ± 0.012	0.559 ± 0.011	0.658 ± 0.007	0.500 ± 0.005
3x-R[b]	0.769 ± 0.013	0.452 ± 0.015	0.492 ± 0.014	0.598 ± 0.006	0.415 ± 0.008
3x-R[c]	**0.817** ± 0.015	0.527 ± 0.015	0.567 ± 0.014	0.661 ± 0.006	0.494 ± 0.005
3x-R*	0.812 ± 0.011	0.523 ± 0.014	0.563 ± 0.013	0.649 ± 0.006	0.493 ± 0.008
3x-Y[a]	0.843 ± 0.029	0.548 ± 0.024	**0.589** ± 0.020	**0.688** ± 0.016	**0.572** ± 0.016
3x-Y[b]	0.803 ± 0.050	0.451 ± 0.035	0.494 ± 0.030	0.623 ± 0.010	0.467 ± 0.017
3x-Y[c]	0.814 ± 0.017	**0.599** ± 0.021	**0.633** ± 0.018	**0.694** ± 0.008	**0.559** ± 0.006
3x-Y*	**0.817** ± 0.037	**0.561** ± 0.033	0.598 ± 0.027	0.685 ± 0.011	0.543 ± 0.014
6x-R[a]	**0.798** ± 0.008	0.558 ± 0.007	0.593 ± 0.006	0.659 ± 0.006	0.507 ± 0.005
6x-R[b]	0.710 ± 0.007	0.501 ± 0.008	0.532 ± 0.008	0.603 ± 0.004	0.423 ± 0.007
6x-R[c]	0.762 ± 0.008	0.571 ± 0.009	0.601 ± 0.008	0.666 ± 0.004	0.499 ± 0.004
6x-Y[a]	**0.785** ± 0.017	0.609 ± 0.015	0.638 ± 0.012	**0.705** ± 0.003	**0.568** ± 0.008
6x-Y[b]	0.725 ± 0.036	0.533 ± 0.021	0.562 ± 0.016	0.635 ± 0.007	0.470 ± 0.172
6x-Y[c]	0.756 ± 0.010	**0.653** ± 0.008	**0.671** ± 0.007	**0.699** ± 0.006	**0.555** ± 0.004
3x-R*+3x-Y*	0.752 ± 0.019	**0.625** ± 0.021	**0.647** ± 0.017	0.686 ± 0.008	0.522 ± 0.004

Supplementary Table S1. Full set of results from Table 3. Precision, recall, and F2 values across all models were measured using an IOU threshold of 0.30 with ground truth. mAP values were calculated using IOU values from 0.25 to 0.75. Bolded values represent the top two scores for each ensemble size and metric. Superscripts *a*, *b*, and *c* represent the type of input processing to train the models as described in the Image Filtering section. Ensembles with * have hybrid inputs, i.e., each ensemble member was trained on a different input processing method.

Models	Avalanche	Precision	Recall	F2	Max F2
1x-R[a]	Conservative	0.530 ± 0.023	**0.730** ± 0.015	0.679 ± 0.010	0.897 ± 0.054
1x-R[b]	Conservative	0.469 ± 0.030	0.695 ± 0.016	0.634 ± 0.018	**0.910** ± 0.067
1x-R[c]	Conservative	0.529 ± 0.026	**0.745** ± 0.012	**0.688** ± 0.012	**0.908** ± 0.046
1x-Y[a]	Posterior	**0.759** ± 0.164	0.647 ± 0.101	0.652 ± 0.051	0.777 ± 0.045
1x-Y[b]	Posterior	0.581 ± 0.206	0.673 ± 0.141	0.620 ± 0.074	0.776 ± 0.090
1x-Y[c]	Posterior	**0.645** ± 0.130	0.724 ± 0.085	**0.695** ± 0.041	0.812 ± 0.056
2x-R[a]	Conservative	0.438 ± 0.011	**0.768** ± 0.012	0.667 ± 0.009	**0.909** ± 0.033
2x-R[b]	Conservative	0.376 ± 0.017	0.743 ± 0.011	0.621 ± 0.013	0.883 ± 0.040
2x-R[c]	Conservative	0.433 ± 0.021	**0.779** ± 0.010	0.671 ± 0.012	**0.909** ± **0.031**
2x-Y[a]	Posterior	**0.634** ± 0.160	0.736 ± 0.083	**0.697** ± 0.028	0.786 ± 0.034
2x-Y[b]	Conservative	0.582 ± 0.103	0.676 ± 0.060	0.648 ± 0.033	0.797 ± 0.081
2x-Y[c]	$\gamma = 0.20$	**0.642** ± 0.065	0.746 ± 0.038	**0.720** ± 0.018	0.807 ± 0.044
3x-R[a]	Conservative	0.394 ± 0.009	0.791 ± 0.010	0.658 ± 0.007	0.895 ± 0.041
3x-R[b]	Conservative	0.331 ± 0.010	0.767 ± 0.012	0.607 ± 0.010	0.886 ± 0.034
3x-R[c]	Conservative	0.385 ± 0.012	**0.797** ± 0.008	0.656 ± 0.008	**0.916** ± 0.028
3x-R*	Conservative	0.340 ± 0.013	**0.809** ± 0.009	0.634 ± 0.011	**0.898** ± 0.026
3x-Y[a]	Posterior	0.558 ± 0.119	0.776 ± 0.056	0.710 ± 0.019	0.786 ± 0.029
3x-Y[b]	Conservative	0.523 ± 0.080	0.728 ± 0.043	0.670 ± 0.016	0.823 ± 0.089
3x-Y[c]	$\gamma = 0.20$	**0.573** ± 0.058	0.780 ± 0.030	**0.725** ± 0.012	0.812 ± 0.036
3x-Y*	Conservative	**0.590** ± 0.069	0.758 ± 0.028	**0.714** ± 0.014	0.802 ± 0.040
6x-R[a]	Conservative	0.320 ± 0.004	0.814 ± 0.006	0.622 ± 0.006	**0.867** ± 0.026
6x-R[b]	Conservative	0.258 ± 0.005	0.802 ± 0.007	0.564 ± 0.007	0.850 ± 0.023
6x-R[c]	Conservative	0.311 ± 0.005	**0.816** ± 0.005	0.616 ± 0.005	**0.912** ± 0.025
6x-Y[a]	$\gamma = 0.20$	**0.536** ± 0.044	0.795 ± 0.022	**0.723** ± 0.010	0.797 ± 0.016
6x-Y[b]	Conservative	0.405 ± 0.028	0.791 ± 0.010	0.664 ± 0.013	0.851 ± 0.030
6x-Y[c]	Conservative	**0.508** ± 0.020	0.797 ± 0.010	**0.715** ± 0.005	0.817 ± 0.026
3x-R*+3x-Y*	Conservative	0.314 ± 0.015	**0.841** ± 0.014	0.630 ± 0.011	0.833 ± 0.052

Supplementary Table S2. Full set of results from Table 4. Precision, recall, and F2 values across all models were measured using an IOU threshold of 0.30 with ground truth. mAP values were calculated using IOU values from 0.25 to 0.75. Bolded values represent the top two scores for each ensemble size and metric. Superscripts *a*, *b*, and *c* represent the type of input processing to train the models as described in the Image Filtering section. Ensembles with * have hybrid inputs, i.e., each ensemble member was trained on a different input processing method.

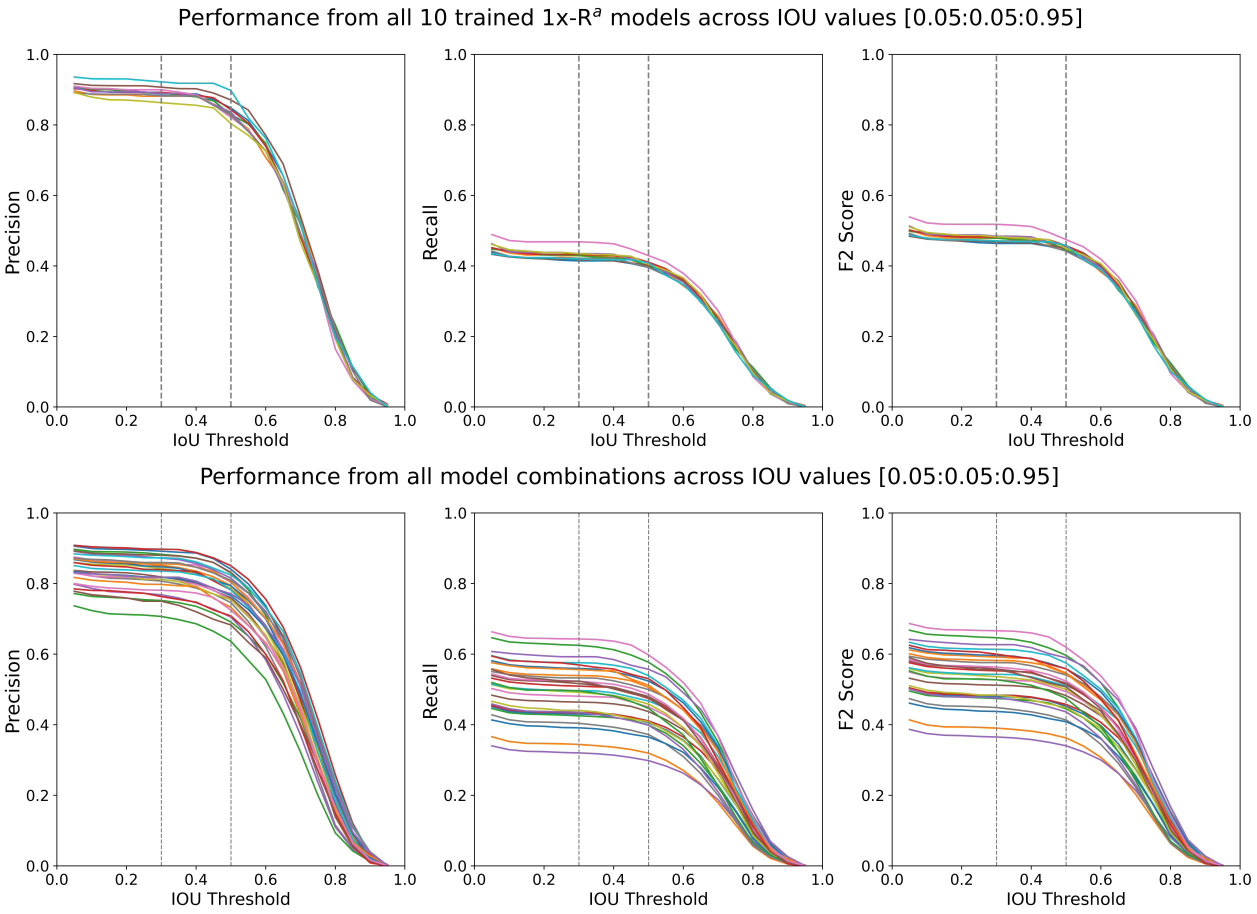

Supplementary Figure S1. Summary of performance metrics versus IOU setting, which dictates whether a proposed region matches an expert labeled region. The top row presents results from single-model RetinaNet and the bottom row presents results from a suite of all different models. For all models, performance is very similar for IOU thresholds ranging from 0.1 to 0.4. We selected an IOU setting of 0.3 as a compromise that is slightly higher in this range requiring more overlap for concordance. It should be noted that unlike many object detection tasks in the computer vision literature, rib fractures generally do not have clear margins leading to clear, unambiguously defined bounds. For this reason, IOU thresholds below the conventional 0.5 setting are warranted.
